# Energy homeostasis deregulation is attenuated by TUDCA treatment in streptozotocin-induced Alzheimer’s disease mice model

**DOI:** 10.1038/s41598-021-97624-6

**Published:** 2021-09-13

**Authors:** Lucas Zangerolamo, Carina Solon, Gabriela M. Soares, Daiane F. Engel, Licio A. Velloso, Antonio C. Boschero, Everardo M. Carneiro, Helena Cristina L. Barbosa

**Affiliations:** 1 Obesity and Comorbidities Research Center, Department of Structural and Functional Biology, University of Campinas, UNICAMP, Campinas, São Paulo CEP: 13083-864 Brazil; 2 Laboratory of Cell Signaling, Obesity and Comorbidities Research Center, University of Campinas, UNICAMP, Campinas, São Paulo Brazil

**Keywords:** Metabolism, Physiology, Neuroscience, Molecular neuroscience

## Abstract

Alzheimer’s disease (AD) is a progressive neurodegenerative disorder and the most common cause of dementia. While cognitive deficits remain the major manifestation of AD, metabolic and non-cognitive abnormalities, such as alterations in food intake, body weight and energy balance are also present, both in AD patients and animal models. In this sense, the tauroursodeoxycholic acid (TUDCA) has shown beneficial effects both in reducing the central and cognitive markers of AD, as well as in attenuating the metabolic disorders associated with it. We previously demonstrated that TUDCA improves glucose homeostasis and decreases the main AD neuromarkers in the streptozotocin-induced AD mouse model (Stz). Besides that, TUDCA-treated Stz mice showed lower body weight and adiposity. Here, we investigated the actions of TUDCA involved in the regulation of body weight and adiposity in Stz mice, since the effects of TUDCA in hypothalamic appetite control and energy homeostasis have not yet been explored in an AD mice model. The TUDCA-treated mice (Stz + TUDCA) displayed lower food intake, higher energy expenditure (EE) and respiratory quotient. In addition, we observed in the hypothalamus of the Stz + TUDCA mice reduced fluorescence and gene expression of inflammatory markers, as well as normalization of the orexigenic neuropeptides AgRP and NPY expression. Moreover, leptin-induced p-JAK2 and p-STAT3 signaling in the hypothalamus of Stz + TUDCA mice was improved, accompanied by reduced acute food intake after leptin stimulation. Taken together, we demonstrate that TUDCA treatment restores energy metabolism in Stz mice, a phenomenon that is associated with reduced food intake, increased EE and improved hypothalamic leptin signaling. These findings suggest treatment with TUDCA as a promising therapeutic intervention for the control of energy homeostasis in AD individuals.

## Introduction

Alzheimer’s disease (AD) is a complex neurodegenerative process and the most common cause of dementia in the elderly, causing major progressive deficits in memory and cognitive function^1^. The pathological hallmarks of AD include extracellular amyloid-β (Aβ) plaques^2^, neurofibrillary tangles (NFTs), composed of hyperphosphorylated and aggregated microtubule-associated protein TAU^3^, neuroinflammation and active gliosis, and significant synaptic and neuronal loss^4^.

A substantial body of evidence suggests that metabolic and non-cognitive abnormalities, such as alterations in neuroendocrine functions, body weight, glucose and energy homeostasis, attributable to hypothalamic dysfunction, are also an integral part of AD, and may contribute to its pathogenesis^5,6^. The hypothalamus orchestrates signals from the brain and the periphery, and controls a range of basic body functions, including feeding behavior and energy balance^7–9^. Hypothalamic dysfunction has often been associated with AD^5,7,10^.

Indeed, abnormal energy metabolism is frequently observed in AD patients and animal models^5,7,10–13^. About 50–60% of AD cases display abnormal eating behaviors^7,14^. Hypothalamic inflammation and increased food intake have already been observed in mice that received intracerebroventricular injection of Aβ oligomers, as well as increased hypothalamic gene expression of orexigenic neuropeptides, suggesting that Aβ oligomers may impair the hypothalamic function involved with satiety^10^. In addition, increased food intake have already been observed in AD animal models, including 3 × TgAD^15,16^, APP/PS1^17^, Tg2576^18^ and transgenic APP23 mice^19^. However, although the literature has addressed selected features of AD attributable to hypothalamic dysfunction, the mechanisms underlying hypothalamus deregulation in AD, especially in the control of energy metabolism, are not yet fully understood.

Currently, several strategies have been proposed for the prevention and treatment of AD and its associated metabolic disorders, among which tauroursodeoxycholic acid (TUDCA) stands out. The bile acid TUDCA is a taurine conjugate of ursodeoxycholic acid (UDCA), abundantly found in bear bile and able to cross the blood–brain barrier^20^. Several studies have shown that TUDCA has neuroprotective action in several models of neurodegenerative disorders, including AD and Parkinson's disease, based on its potent ability to inhibit apoptosis, attenuate oxidative stress, and reduce endoplasmic reticulum (ER) stress^20^. It is known that TUDCA reduces the accumulation of βA plaques in transgenic APP/PS1 AD mice model, in addition to decreasing the activation of astrocytes and microglia, which results in attenuation of neurotoxic potential and inflammatory response, as well as in decreased loss of neuronal integrity, altogether resulting in improved cognitive ability^21–23^.

In a recent study, we explored the effects of TUDCA upon glucose metabolism in an experimental AD mouse model^24^. Using the streptozotocin-induced AD mice model (Stz), which exhibits structural, neurochemical and behavioral changes that mimic human sporadic AD^24,25^, we observed that these mice treated with TUDCA showed higher glucose tolerance and insulin sensitivity, reduced fasted and fed glycemia, increased islet mass and beta cell area, as well as potentiated glucose-stimulated insulin secretion, compared with Stz group treated with PBS. In addition, we also observed that body weight and fat pad deposits were lower in Stz animals that received TUDCA, suggesting that this bile acid can also modulate energy homeostasis in this model.

Here, as a follow-up of our previous report^24^, we explored factors potentially involved in the reduced body weight and adiposity observed in Stz mice after TUDCA treatment. Considering that body weight is mainly regulated by food intake and energy expenditure (EE)^26^, we wanted to explore the mechanisms involved in TUDCA’s actions that resulted in this phenotype. We hypothesized that the beneficial effects of TUDCA upon the energy metabolism of Stz mice could be the result of one or more of the following mechanisms: *i*, changes in food behavior, leading to reduced food intake; *ii*, changes in hypothalamus function, modulating the expression of proinflammatory markers and neuropeptides, as well as EE; *iii*, improvement in hypothalamic leptin signaling; and *iv*, an increase in thermogenesis in brown adipose tissue (BAT).

## Material and methods

### Experimental animals

For all experiments, 2-month-old male C57BL/6 mice were purchased from the animal facility of the University of Campinas. Mice were maintained on a 12-h light–dark cycle, in a temperature-controlled facility with free access to food and water. The Ethics Committee at the University of Campinas (protocol numbers: 4698-1/2017 and 5022-1/2018) approved all experimental procedures involving mice, which were conducted in accordance to the last revision of the National Institutes of Health (NIH) guide for the care and use of laboratory animals. The study was carried out in compliance with the ARRIVE guidelines.

### Stereotaxic surgery

Mice were submitted to intracerebroventricular (ICV) injection in the lateral ventricles under xylazine (10 mg/kg) and ketamine (100 mg/kg) anesthesia, using a Stoelting (Wood Dale, IL, USA) stereotaxic apparatus. The stereotaxic coordinates relative to the Bregma were: anteroposterior: − 0.5 mm; lateral: ± 1.1 mm, and depth: − 2.8 mm, based on the previously published method^24,27^. The injection of Evan blue and dissection of the region of interest provided the anatomical control of stereotaxic procedure. Streptozotocin (3 mg/kg) was injected bilaterally into the lateral ventricles to generate the streptozotocin-induced Alzheimer’s disease mice model (Stz). Control mice (Ctl) received vehicle injection (citrate buffer 0.05 mol/L, pH 4.5), in the same coordinates. A total of 1.5 μL of streptozotocin or citrate buffer was infused in each lateral ventricle at a rate of 0.5 μL/min. The injection was repeated 2 days after the first streptozotocin injection (1.5 mg/kg per day of injection).

### TUDCA treatment

Six days after the first application of streptozotocin, part of the animals that received this drug were randomly selected to be treated with the bile acid TUDCA. TUDCA (Calbiochem, Sao Paulo, Brazil; cat. 580,549) was dissolved in phosphate buffer saline (PBS), and was intraperitoneally (i.p.) injected. TUDCA was daily injected (1 injection/day), for 10 consecutive days, at a dose of 300 mg/Kg body weight, based on previous studies^24,28,29^. The rest of the mice, as well as the control mice, received PBS alone instead of TUDCA. Thus, the 3 experimental groups used in this work were formed: *i*) mice that received ICV injection of citrate buffer and treated with PBS for 10 consecutive days (Ctl group); *ii*) mice that received ICV injection of streptozotocin and treated with PBS for 10 consecutive days (Stz group), and *iii*) mice that received ICV injection of streptozotocin and treated with TUDCA for 10 consecutive days (Stz + TUDCA group). In all experiments, control and intervention group mice were treated in the same experimental settings.

### Food intake assessment

During the 10-days of treatment with TUDCA or PBS, mice were housed individually in home cages, and the food intake was evaluated every 2 days. A known amount of diet was placed in each cage, and the determination of the food consumption on days 2, 4, 6, 8 and 10 was assessed at 7 a. m.

### Energy expenditure, respiratory quotient, and locomotor activity

At the end of TUDCA treatment, a group of mice was individually placed in sealed metabolic cages, and after 24 h of acclimatization, O_2_ consumption and CO_2_ production were measured for 24 h (Oxylet system; Pan Lab/Harvard Instruments, Barcelona, Spain). Respiratory quotient (RQ) and EE were then calculated using the O_2_ and CO_2_ data by Metabolism® Software (Pan Lab/Harvard Instruments)^30^. For locomotor activity detection, mice were individually placed in cages (Multitake Cage LE001 PH; Pan Lab/Harvard Instruments), and then acclimated for 24 h. After acclimatization, the locomotor activity (in the xy- and z-axes) was registered using Compulse® and Actitrack software (Pan Lab/Harvard Instruments) for the next 24 h.

### Serum leptin measurement

To obtain serum, blood samples were centrifuged (1100 g for 15 min at 4 °C), and serum were stored at − 80 °C for posterior leptin quantification. Leptin concentration was measured using specific commercial enzyme-linked immunosorbent assay (ELISA) kit (Chrystal Chem, Inc, Downers Grove, IL, USA; cat No. #90030), according to the manufacturer's instructions.

### Leptin sensitivity

At the end of TUDCA treatment, mice were fasted overnight (12 h) and given intraperitoneal leptin (5 mg/kg) (recombinant mouse leptin from ©Merck-Calbiochem cat. #429705, Darmstadt, Germany). A known amount of food was added to each cage and food intake were then measured 1, 2, 4, 12, and 24 h after leptin injection.

To evaluate leptin activation of its intracellular signaling pathway, JAK2 and STAT3 phosphorylation was assessed after an acute leptin stimulus (5 mg/kg)^31^. Fasted mice (12 h) received an intraperitoneal injection of leptin and were euthanized 45 min later. Hypothalamus were collected and stored at − 80 °C for p-JAK2 and p-STAT3 western blot analysis.

### Body parameters and tissue collection

The body weight of all mice was evaluated prior to the start of TUDCA or PBS treatment. At the end of TUDCA treatment, fasting mice were weighed and weight gain during the experimental period was calculated. Then, mice were euthanized by decapitation (for blood collection), after inhalation of isoflurane. The hypothalamus and interscapular BAT were collected, immediately frozen in liquid nitrogen, and stored at − 80 °C until analysis. Furthermore, the epididymal and retroperitoneal adipose tissue, as well as gastrocnemius skeletal muscle were dissected and weighed.

### RNA extraction and real-time qPCR

The total RNA content of the hypothalamus and BAT samples was extracted and quantified as previously described^32^. To prepare the cDNA, 1 μg of total mRNA and High-Capacity cDNA Reverse Transcription Kit (Applied Biosystems™, Thermo Fisher Scientific Inc, Waltham, MA, USA) were used. Real time PCR was performed on 7500 Fast Real-time PCR System (Applied Biosystems™) using Fast SYBR® Green Master Mix (Applied Biosystems™). The specificities of amplifications were confirmed by melting-curve analyses. The relative expression of mRNAs was calculated after normalization with GAPDH, using the 2^−ΔΔCT^ method. The primer sequences were designed and purchased from IDT®-Integrated DNA Technologies, and are shown in Table 1.

**Table 1:** Primer sequences for real-time qPCR assays.

Gene	Forward (5′–3′)	Reverse (5′–3′)
TNF-α	CCCTCACACTCAGATCATCTTCT	GCTACGACGTGGGCTACAG
IL-1β	GCAACTGTTCCTGAACTCAACT	ATCTTTTGGGGTCCGTCAAC
IFN-γ	ATGAACGCTACACACTGCATC	CCATCCTTTTGCCAGTTCCTC
GFAP	CCCTGGCTCGTGTGGATTT	GACCGATACCACTCCTCTGTC
POMC	GGCTTGCAAACTCGACCTC	TGACCCATGACGTACTTCCG
CART	ACCTTTGCTGGGTGCCCGTG	TGCAACGCTTCGATCAGCTCC
AgRP	GAGTTCCCAGGTCTAAGTCTGAATG	ATCTAGCACCTCCGCCAAAG
NPY	TACTCCGCTCTGCGACACTA	TCTTCAAGCCTTGTTCTGGG
DIO2	AATTATGCCTCGGAGAAGACCG	GGCAGTTGCCTAGTGAAAGGT
PPARGC1α	TATGGAGTGACATAGAGTGTGCT	CCACTTCAATCCACCCAGAAAG
CIDEA	TGCTCTTCTGTATCGCCCAGT	GCCGTGTTAAGGAATCTGCTG
PRDM16	TGCTGACGGATACAGAGGTGT	CCACGCAGAACTTCTCGCTAC
COX7A1	CAGCGTCATGGTCAGTCTGT	AGAAAACCGTGTGGCAGAGA
COX8B	TGTGGGGATCTCAGCCATAGT	AGTGGGCTAAGACCCATCCTG
UCP1	CTGCCAGGACAGTACCCAAG	TCAGCTGTTCAAAGCACACA
Lep-R	TGGTCCCAGCAGCTATGGT	ACCCAGAGAAGTTAGCACTGT
GAPDH	AGGTCGGTGTGAACGGATTTG	AGTAGACCATGTAGTTGAGGTCA

*TNF-α* tumor necrosis factor alpha; *IL-1β* interleukin 1 beta; *IFN-γ* interferon gamma; *GFAP* glial fibrillary acidic protein; *POMC* proopiomelanocortin; *CART* cocaine and amphetamine regulated transcript; *AgRP* agouti-related peptide; *NPY* neuropeptide Y; *DIO2* iodothyronine deiodinase 2; *PPARGC1α* peroxisome proliferator-activated receptor gamma coactivator 1 alpha; *CIDEA* cell death-inducing DNA fragmentation factor alpha-like effector A; *PRDM16* PR/SET domain 16; *COX7A1* cytochrome c oxidase subunit 7A1, *COX8B* cytochrome c oxidase subunit 8B, *UCP1* uncoupling protein 1; *Lep-R* leptin receptor; *GAPDH* glyceraldehyde 3-phosphate dehydrogenase.

#### Western blot analysis

Western blot analysis was performed as previously described^33^. Briefly, hypothalamus and BAT samples were homogenized in lysis buffer and centrifuged (12,000 g for 20 min at 4 °C) to obtain a protein extract. Protein concentration was determined by the Bradford method, using bovine serum albumin as standard curve. Thirty μg of the protein samples were homogenized and boiled in a Laemmli buffer, and then proteins were separated by 10–12% SDS-PAGE. The transfer to nitrocellulose membranes was performed in a Trans Blot transfer with tris/glycine buffer. After, the membranes were blocked with 5% BSA for 1 h at room temperature, and were then incubated overnight at 4 °C with specific antibodies —p-JAK2 (Tyr1007/Tyr1008) (Santa Cruz Biotechnology cat. 16566-R), total JAK2 (Santa Cruz Biotechnology cat. 278), p-STAT3 (Tyr705) (Cell Signaling cat. 9131), total STAT3 (Cell Signaling cat. 4904), and UCP1 (Cell Signaling cat. 14670)— that were diluted 1:1000. GAPDH (Sigma Aldrich, cat. G9545) was used as an internal control of the experiment. After incubation, the appropriate secondary antibody (dilution 1:10,000; Invitrogen, Sao Paulo, Brazil) was added and subsequently detected by exposure to chemiluminescent substances (luminol and peroxidase) in Amersham Imager 600 (GE Healthcare Life Sciences, Buckinghamshire, UK). The quantification of the bands was performed by densitometry using the ImageJ software (National Institutes of Health, Bethesda, MD, USA).

#### Heart perfusion and hypothalamic immunostaining

At the end of TUDCA treatment, a group of mice was deeply anesthetized with ketamine and xylazine, and perfused with an intracardiac infusion of 0.9% saline, followed by 4% formaldehyde. The brain was removed from each mouse, post-fixed for 24 h in 4% paraformaldehyde (PFA) solution at room temperature, and cryoprotected by immersion in a buffer containing 30% sucrose and then 40% at 4 °C. A series of 20 μm-thick frozen sections were prepared using a cryostat (LEICA Microsystems®, CM1860, Buffalo Grove, IL, USA) and stored in an anti-freezing solution. For the immunostaining, sections were first blocked using 10% goat serum (GS) diluted in PBS containing 2% Triton X-100 for 2 h at room temperature. Sections were then incubated overnight at 4 °C in rabbit anti-Iba-1 primary antibody (1:200; Wako LKN4881) in a blocking solution. After washing in PBS, tissue sections were incubated with anti-rabbit FITC (1:200; Santa Cruz sc2012) in a blocking solution for 2 h at room temperature. After that, the sections were washed in PBS and then incubated with 0.5 g/mL 4′,6-diamidino-2-phenylindole (DAPI, Invitrogen, D1306) for 10 min, washed, mounted in Vectashield (Vector, H-1200) and covered with coverslips. Finally, sections were visualized using a confocal microscope (Upright LSM780-NLO Zeiss) and fluorescence intensity was quantified with ImageJ software (National Institutes of Health, Maryland, USA).

#### Statistical analysis

Results are displayed as the mean ± standard error of the mean (SEM). The data were analyzed by one-way analysis of variance (ANOVA) followed by the Tukey post-hoc-test, using GraphPad Prism version 6.00 software (GraphPad Inc., CA, USA). A p value ≤ 0.05 was considered significant.

## Results

### Mice treated with TUDCA present lower body weight and fat pads depots, compared with Stz mice

**Figure 1:**
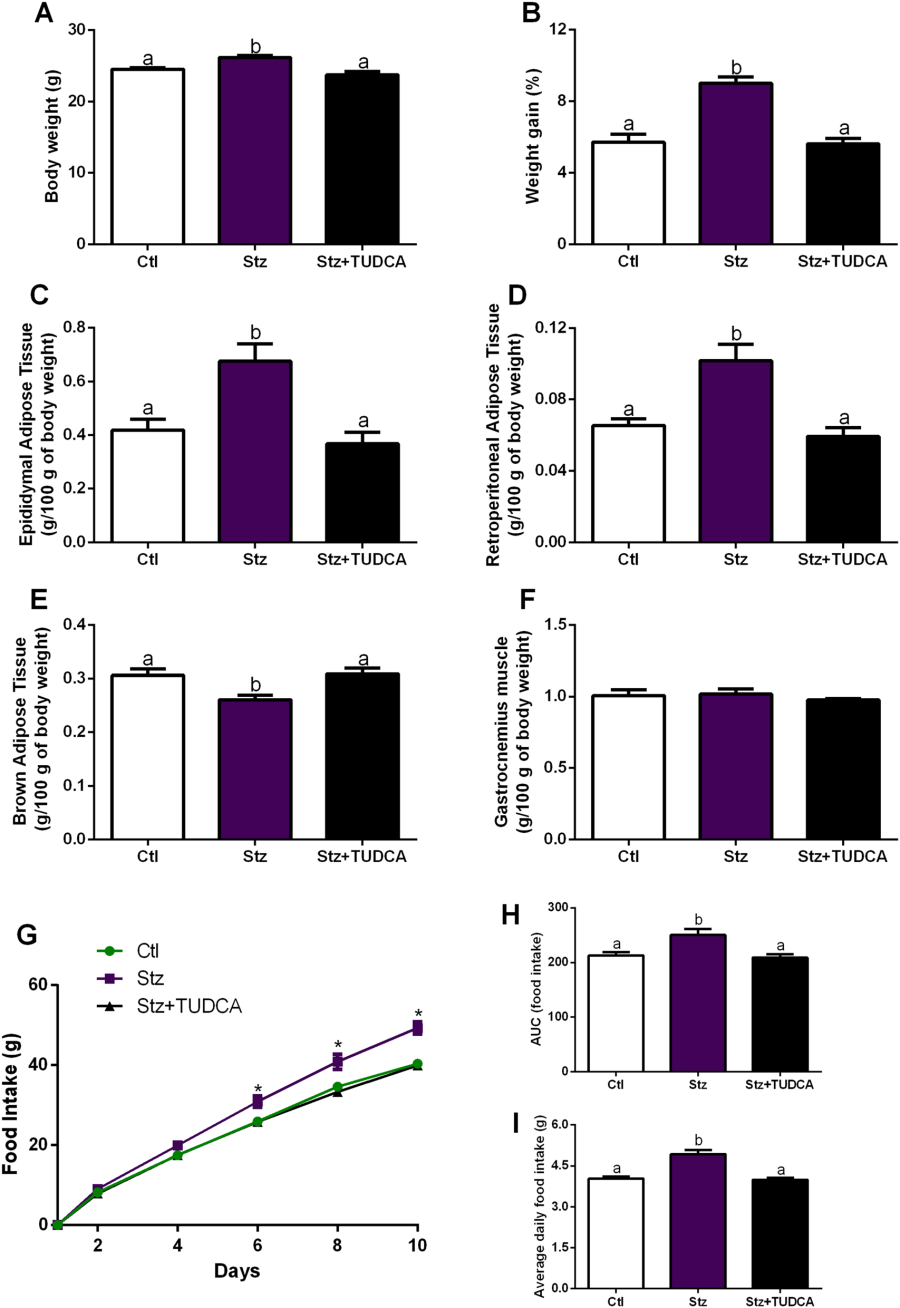
Mice treated with TUDCA display lower body weight, adiposity and food intake. Body weight (**A**) and percent body weight gain (**B**). Epididymal (**C**), retroperitoneal (**D**) and interscapular brown adipose tissue (**E**) fat pad weight. Gastrocnemius weight (**F**). Cumulative food intake over 10 days (**G**), AUC of 10-days food intake (**H**) and average daily food intake (**I**). Data are expressed as means ± SEM (n = 6–8). Different letters indicate significant differences between groups and * indicates Stz is different from Ctl and Stz + TUDCA mice (*P* ≤ 0.05), based on one-way ANOVA test followed by Tukey post-hoc-test.

Stz mice displayed increased body weight at the time of euthanasia, as well as higher percentage of body weight gain during the experimental period (Fig. 1A,B). Consistent with these findings, Stz mice presented increased epididymal and retroperitoneal fat accumulation compared with Ctl group (Fig. 1C,D). In addition, we also observed lower BAT weight in Stz mice (Fig. 1E). However, Stz + TUDCA mice showed lower body weight, weight gain, and fat accumulation, as well as higher BAT weight in comparison with those observed in Stz mice (Fig. 1A–D). Regarding the skeletal muscle pad, no differences were observed between the groups (Fig. 1F).

### Mice treated with TUDCA present lower food intake, compared with Stz mice

To access food intake, mice were kept individualized in cages, and food intake was assessed during the 10-days of treatment with TUDCA or PBS. As noted in Fig. 1G–I, Stz mice showed higher food intake than did Ctl mice, as determined by the AUC of food intake (Fig. 1H), as well as the higher average daily food intake (Fig. 1I). In contrast, after 6 days of TUDCA treatment, the cumulative food intake curve for Stz + TUDCA group was below the respective untreated group (Fig. 1G), showing a clear significant effect at the end of the 10-days of treatment.

### Mice treated with TUDCA present higher RQ and EE, compared with Stz mice

**Figure 2:**
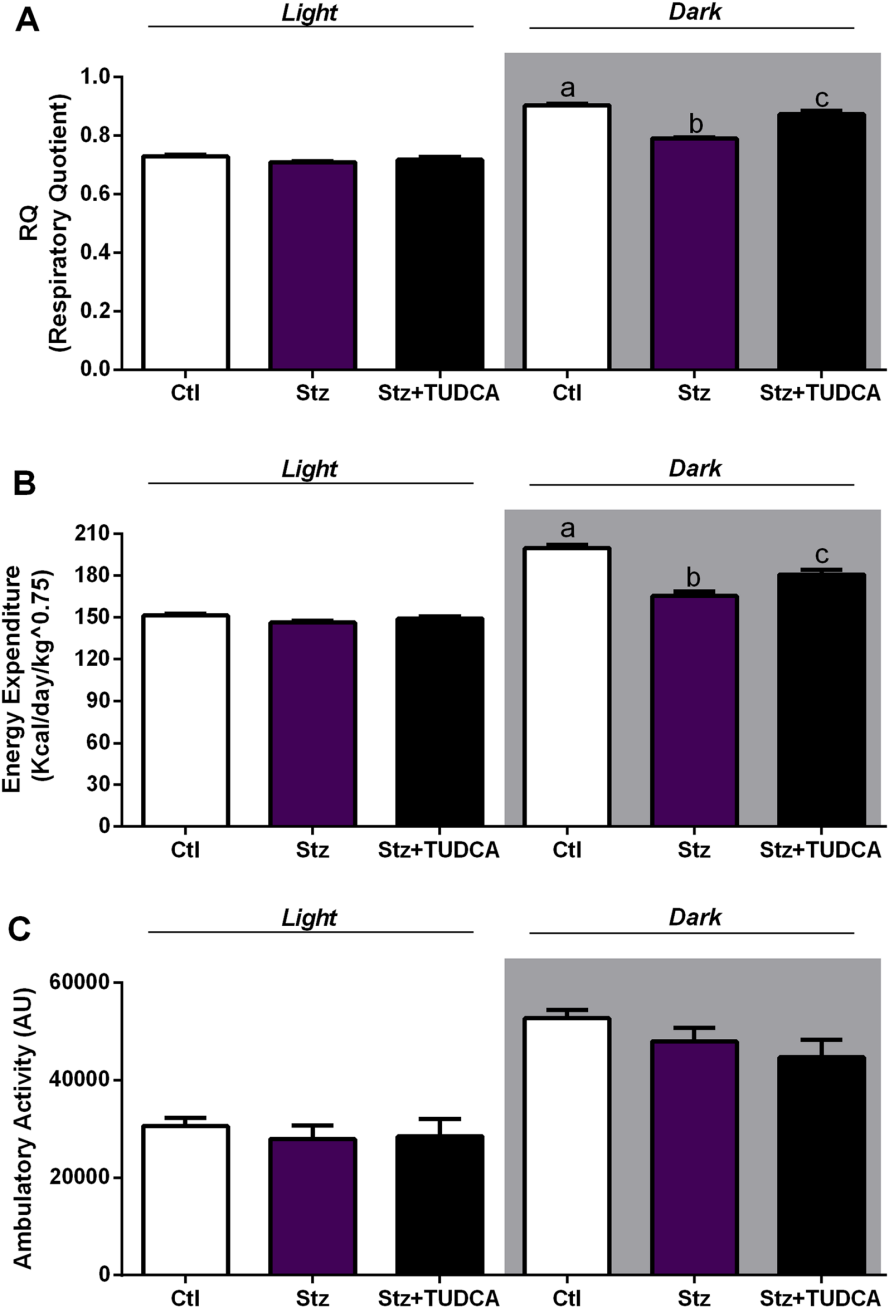
Mice treated with TUDCA display higher RQ and EE. Respiratory quotient (RQ) (**A**), energy expenditure (EE) (**B**) and locomotor activity (**C**) average during light (7 a.m. to 6:59 p.m.) and dark (7 p.m. to 6:59 a.m.) cycles. Data are expressed as means ± SEM (n = 4–6). Different letters indicate significant differences between groups (*P* ≤ 0.05), based on one-way ANOVA test followed by Tukey post-hoc-test. *AU* arbitrary units.

The analyses of RQ values showed that Stz mice presents impairment in the metabolic flexibility, as judged by their incapacity to efficiently increase the RQ values during the dark cycle (feeding period), as the Ctl animals (Fig. 2A), evidencing a lower carbohydrate oxidation capacity. On the other hand, Stz + TUDCA mice displayed increased values of RQ during the dark cycle, similar to those observed in Ctl group, indicating higher carbohydrate oxidation (Fig. 2A). To evaluate whether this metabolic shift would affect energy metabolism, we assessed the EE in the animals. Analyses of EE over 24 h showed that Stz mice exhibited reduced EE in the dark cycle (Fig. 2B). Otherwise, Stz + TUDCA mice showed higher EE during the dark cycle, compared with Stz group (Fig. 2B). To support this outcome, we investigated the association between the higher EE found in Stz + TUDCA mice and spontaneous locomotor activity. However, no differences in locomotor activity were observed between the groups (Fig. 2C).

### Mice treated with TUDCA present reduced proinflammatory markers and orexigenic neuropeptides in the hypothalamus, compared with Stz mice

**Figure 3:**
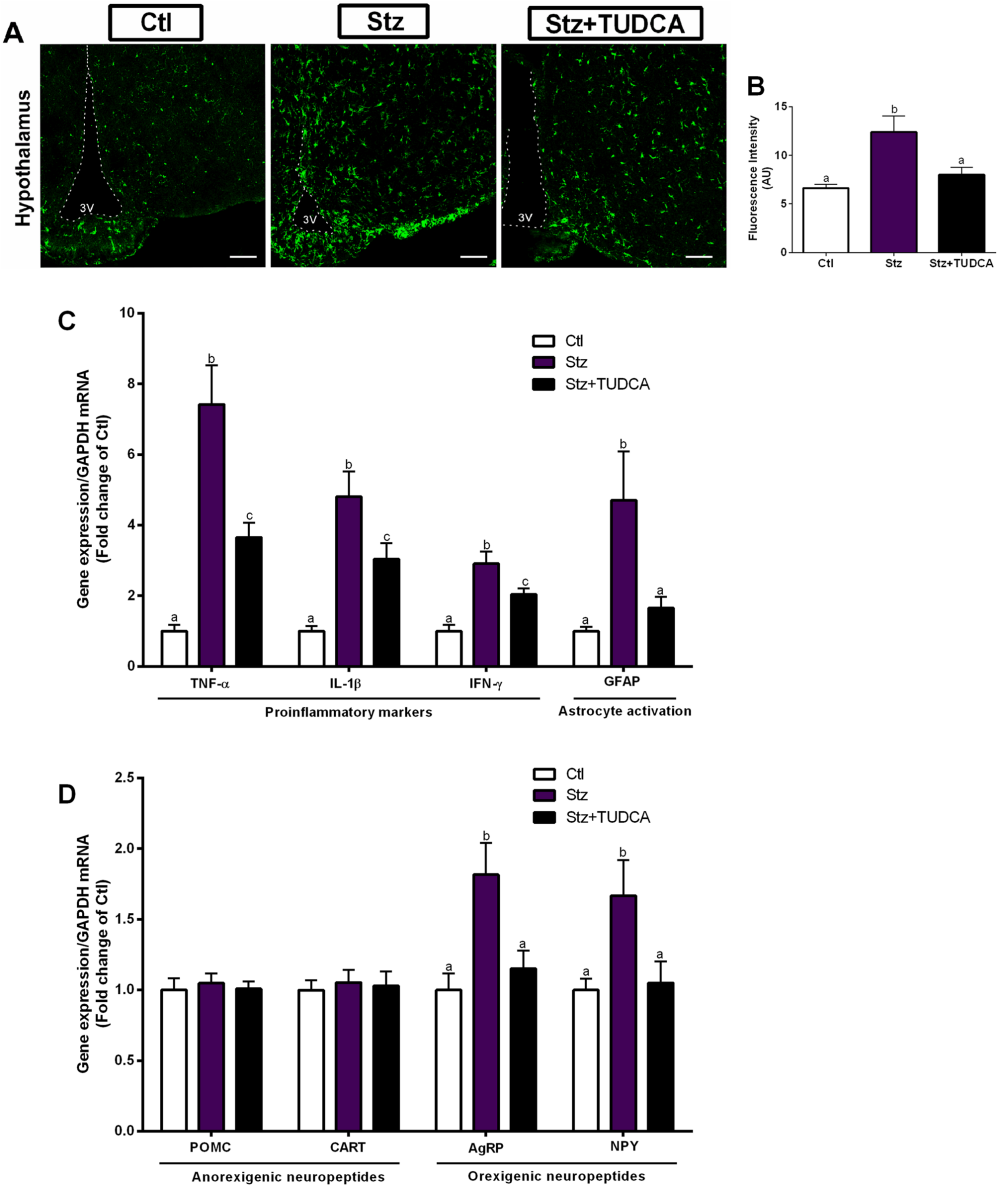
Mice treated with TUDCA display reduced gene expression of proinflammatory markers and orexigenic neuropeptides in the hypothalamus. Iba-1 immunoreactivity (green), scale bars 100 μm, dotted lines mark the 3 V walls (**A**) and quantification of Iba-1 positive staining in the hypothalamus (**B**). Real-time PCR analysis of hypothalamic TNF-α, IL-1β, IFN-γ and GFAP mRNA expression (**C**) normalized by GAPDH. Real-time PCR analysis of hypothalamic POMC, CART, AgRP and NPY mRNA levels (**D**) normalized by GAPDH. Data are expressed as means ± SEM (n = 5–8). Different letters indicate significant differences between groups (*P* ≤ 0.05), based on one-way ANOVA test followed by Tukey post-hoc-test. 3 V: third ventricle, *AU* arbitrary units.

Next, we went to evaluate the animals' hypothalamus, since it is directly involved with feeding behavior and energy homeostasis. First, we quantified the fluorescence intensity of Iba-1, a common marker of microglia activation^34^. We found an increase in the fluorescence intensity of Iba-1 in the hypothalamus of the Stz mice, while the Stz + TUDCA mice showed Iba-1 fluorescence statistically similar to those observed in the Ctl mice (Fig. 3A,B). As increased microglia activation is usually associated with increased proinflammatory markers production^35^, we next investigated weather higher Iba-1 fluorescence intensity found in Stz mice would result in increased expression of proinflammatory genes in the hypothalamus. As expected, Stz mice showed higher gene expression of inflammatory markers, such as TNF-α, IL-1β, IFN-γ (Fig. 3C). In contrast, when compared with Stz group, mice treated with TUDCA showed reduced expression of these genes (Fig. 3C). The hypothalamic gene expression of GFAP, a marker of reactive astrocytes, has also been evaluated in animals, since reactive astrocytes can release a wide range of inflammatory mediators in the brain^36,37^. An increase in GFAP mRNA levels was detected in Stz mice. However, this increase was not observed in Stz + TUDCA mice (Fig. 3C). Then, a question was raised whether the proinflammatory condition observed in Stz group would be modulating the expression of hypothalamic neuropeptides. Consistent with increased food ingestion observed in Stz mice, elevated hypothalamic expression of orexigenic neuropeptides AgRP and NPY (but no alterations in anorexigenic POMC and CART mRNA levels) was also detected in Stz mice (Fig. 3D). On the other hand, the treatment with TUDCA prevented the increase in gene expression of these neuropeptides, involved in stimulating food intake (Fig. 3D).

### Mice treated with TUDCA present ameliorated hypothalamic leptin signaling, compared with Stz mice

**Figure 4:**
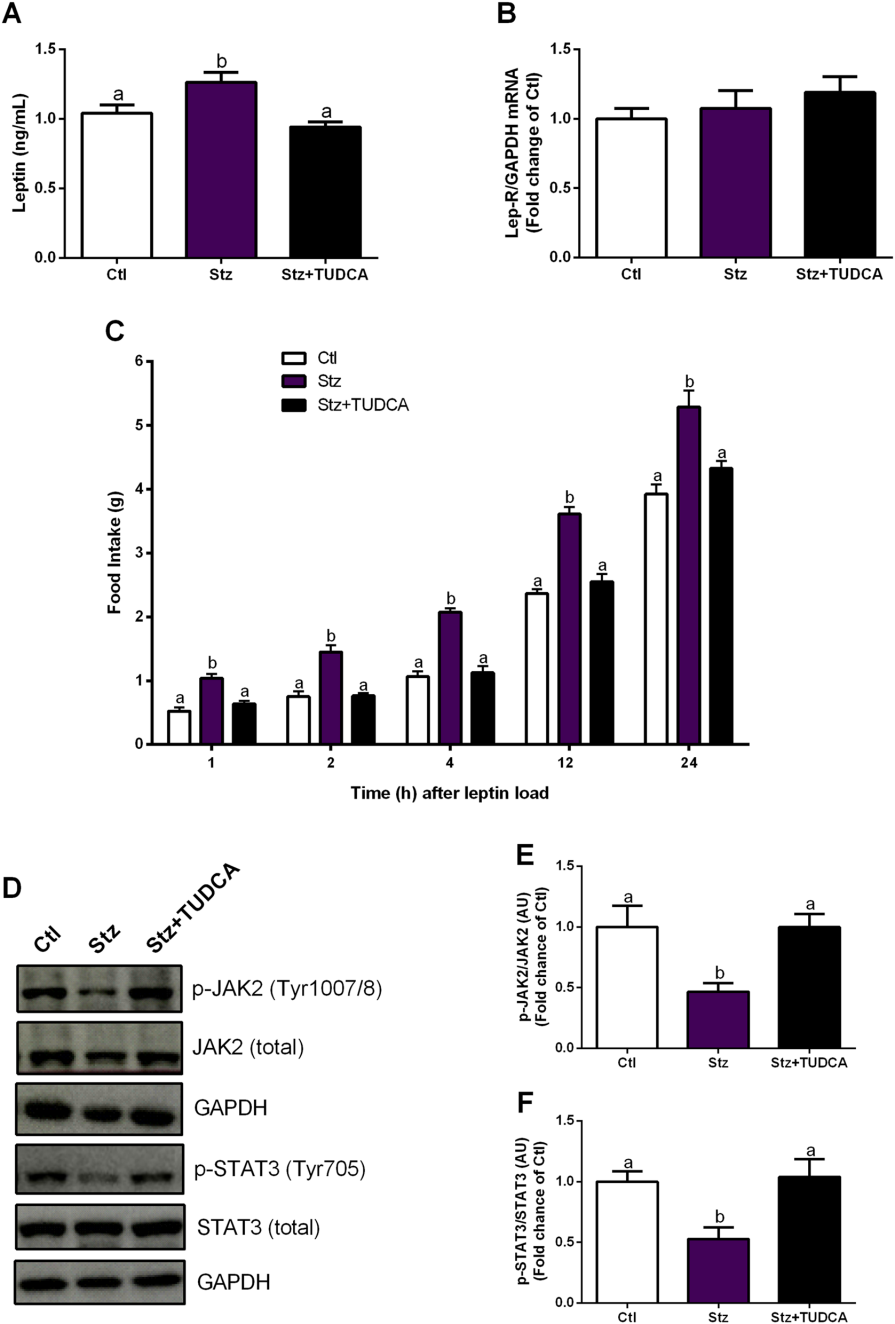
Mice treated with TUDCA display ameliorated hypothalamic leptin signaling. Serum leptin levels (**A**) and gene expression of Lep-R (**B**) normalized by GAPDH in the hypothalamus. Acute food intake after leptin (5 mg/kg, i.p.) load (**C**). Representative western blot images of p-JAK2, total JAK2, p-STAT3, total STAT3 and GAPDH in the hypothalamus (**D**). Protein content of p-JAK2 (Tyr1007/8) normalized by total JAK2 (**E**) and p-STAT3 (Tyr705) normalized by total STAT3 (**F**) in the hypothalamus, 45 min after leptin (5 mg/kg, i.p.) load. GAPDH was employed as a housekeeping protein for total JAK2 and STAT3 normalization. Data are expressed as means ± SEM (n = 4–8). Different letters indicate significant differences between groups (*P* ≤ 0.05), based on one-way ANOVA test followed by Tukey post-hoc-test. *AU* arbitrary units.

In order to provide a more comprehensive view of the energy metabolism in Stz mice, we also measured serum leptin concentration, as well as leptin receptor mRNA levels in the hypothalamus. As expected, Stz mice exhibited higher levels of serum leptin, since the secretion of this hormone is proportional to the fat mass^38^, while TUDCA treated group displayed leptin levels similar to those observed in Ctl mice (Fig. 4A). Regarding the Lep-R gene expression, no statistical changes were observed between the groups (Fig. 4B). Next, to determine whether the increased sign of inflammation observed in the hypothalamus of Stz group impaired the leptin's ability to trigger a hypothalamic anorexigenic response, food intake was measured following an acute i.p. injection of leptin. Significantly, Stz mice failed to exhibit the expected suppression in acute food intake upon i.p. administration of leptin, suggesting central leptin resistance (Fig. 4C). On the other hand, Stz + TUDCA mice displayed food intake statistically similar to those observed in Ctl mice (Fig. 4C). To confirm the finding regarding leptin resistance suggested by the previous result, we analyzed the hypothalamic leptin activation of its intracellular signaling pathway, through JAK2 and STAT3 phosphorylation. Indeed, after an acute i.p. injection of leptin, we observed lower JAK2 and STAT3 phosphorylation levels in Stz mice (Fig. 4D–F). However, Stz + TUDCA mice displayed higher levels of p-JAK2 and p-STAT3 than Stz mice, presenting values similar to those observed in Ctl group (Fig. 4D–F). Taken together, these data indicate that leptin signaling is disrupted in Stz group, while the treatment with TUDCA preserved the signaling of this hormone in Stz + TUDCA animals.

### TUDCA treatment does not modulate the expression of thermogenesis markers in BAT of Stz mice

**Figure 5:**
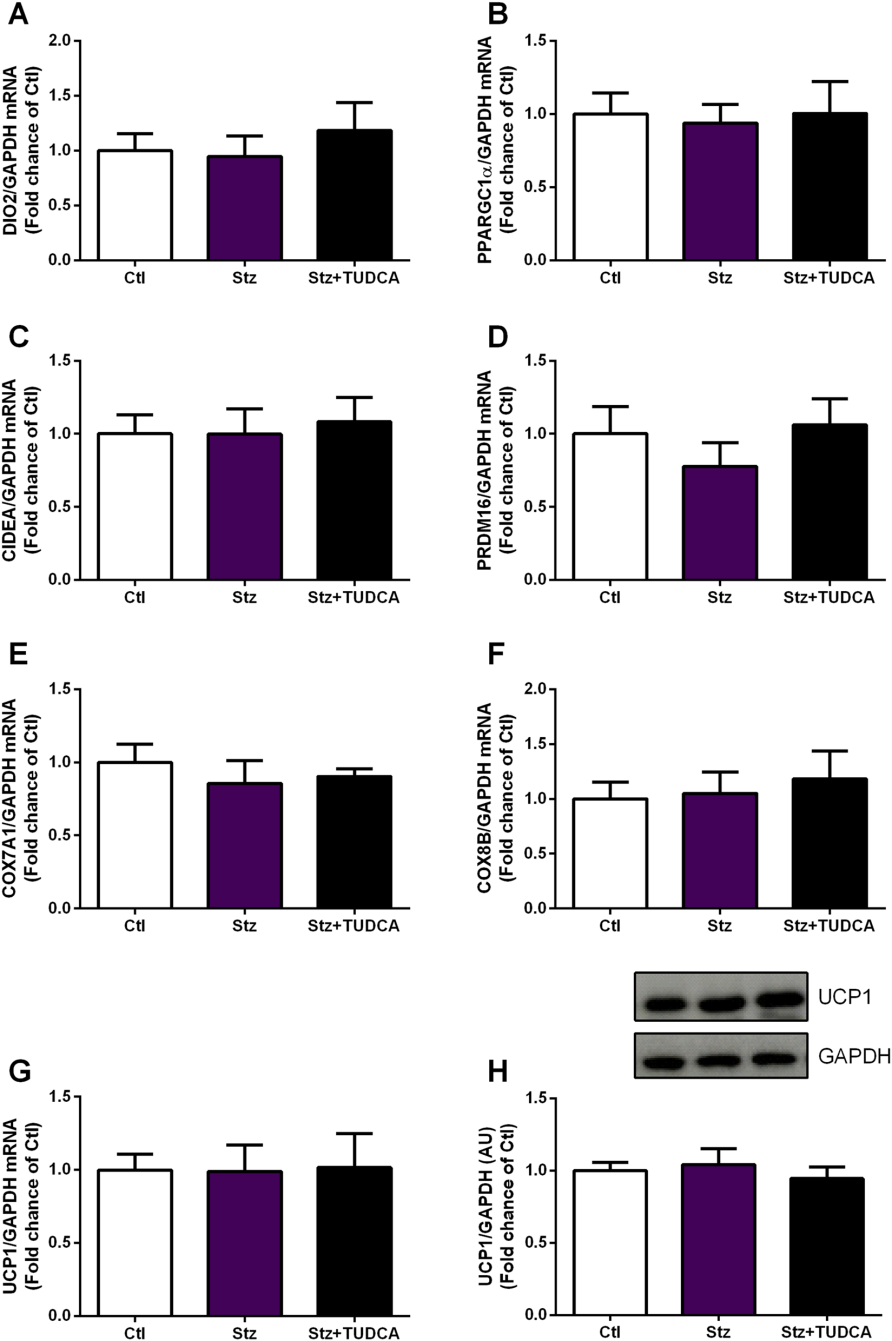
TUDCA treatment does not modulate the expression of thermogenesis markers in BAT. Real-time PCR analysis of DIO2 (**A**), PPARGC1α (**B**), CIDEA (**C**), PRDM16 (**D**), COX7A1 (**E**), COX8B (**F**) and UCP1 (**G**) normalized by GAPDH in the brown adipose tissue. Protein content of UCP1 (H) normalized by GAPDH in the brown adipose tissue. Data are expressed as means ± SEM (n = 7–8). No significant differences between groups were observed (*P* ≤ 0.05), based on one-way ANOVA test followed by Tukey post-hoc-test. AU: arbitrary units.

Finally, to investigate whether the effects of TUDCA on energy homeostasis may also be dependent on modulations in the thermogenic capacity of BAT, we quantified the mRNA levels of thermogenesis-related genes in this tissue, as well as the gene expression and protein content of UCP1, considered the main thermogenesis marker in adipocytes^39^. However, no significant changes were detected in the gene expression of DIO2, PPARGC1α, CIDEA, PRDM16, COX7A1 and COX8B (Fig. 5A–F). In addition, no difference between groups was detected in terms of gene expression and protein content of UCP1 in the BAT (Fig. 5G,H).

## Discussion

AD is a complex progressive neurodegenerative process, which leads to decrease in cognitive function^1^. However, several studies have reported that metabolic and non-cognitive abnormalities, such as alterations in glucose and energy homeostasis, attributable to hypothalamic dysfunction, are also an important part of AD, and may contribute to its pathogenesis^5,6,10,17,19,24^. Although evidence pointing to hypothalamic dysfunction has long been provided, there has been a relative lack of attention to the hypothalamus in AD research^5^. Several autopsy studies have reported Aβ plaques and NFTs in the hypothalamus of AD subjects^5^. Moreover, it is known that diverse hypothalamic nuclei are affected in AD^40,41^. Consistent with these findings, significant atrophy has been identified in the hypothalamus of AD patients^42,43^, evidencing that, in fact, the hypothalamus is clearly impaired in AD.

Thus, there is a search for molecules that could improve cognitive and metabolic deregulation in AD and contribute to the reduction of the deleterious secondary effects mentioned above. In this context, the bile acid TUDCA has emerged as an important candidate due to its neuroprotective action^20^. We recently reported that TUDCA treatment normalized AD pathological neuromarkers in streptozotocin-induced AD mouse model. Besides, we also showed that Stz mice treated with TUDCA presented improvement in glucose-insulin homeostasis and reduced fat pad depots, as well as in body weight^24^, suggesting that this bile acid can also modulate energy homeostasis in this model.

Taking this into account, we used the streptozotocin-induced AD mice model treated with TUDCA, and investigated factors potentially involved in the reduced body weight and adiposity observed in these animals. Considering that body weight is mainly regulated by food intake and EE^26,44^, we firstly evaluated these two factors. We observed an increase in food intake and a reduction in EE in Stz mice, while both parameters were improved after treatment with TUDCA.

It is well established that body weight and appetite control are complex and dependent on the balance between the expression of specific genes in the hypothalamus^7^. Within the hypothalamus, more specifically in the arcuate nucleus, there are two sets of neurons involved in the regulation of eating behavior: neuropeptide Y (NPY) neurons, that coexpress agouti-related transcript (AgRP); and proopiomelanocortin (POMC) neurons, that coexpress cocaine-amphetamine-related transcript (CART), which seem to be the main targets of leptin action. NPY/AgRP activation stimulates food intake (orexigenic effect), while POMC/CART activation inhibits food intake (anorexigenic effect)^44^.

In agreement with the increased food intake observed in the animals of the Stz group, we also observed in these animals a high hypothalamic expression of the orexigenic neuropeptides AgRP and NPY, without alterations in the gene expression of the anorexigenic neuropeptides POMC and CART. Consistent with our data, increased gene expression of orexigenic neuropeptides was also observed in the hypothalamus of mice that received ICV injection of βA oligomers^10^.

The gradual accumulation of Aβ peptide in AD brains induces inflammatory reactions, in which activated microglial cells are mostly involved^45^. Activated microglia, as well as reactive astrocytes, are frequently found close to the βA plaques, evidencing the participation of these cells in neuroinflammation and neurodegeneration^36,46^. Remarkably, there is an extensive literature documenting the role of βA as a potent microglia activator, with the presence of constant βA plaques can keep microglia continuously activated, resulting in the production of proinflammatory cytokines and chemokines and leading to chronic CNS inflammation^35^. In addition, reactive astrocytes induced by activated microglia will also contribute to the secretion of proinflammatory factors^36^.

In fact, we observed an inflammatory condition in Stz mice, evidenced by the high Iba-1 fluorescence intensity and expression of proinflammatory genes in the hypothalamus. On the other hand, these inflammatory markers were reduced in Stz + TUDCA mice, compared to Stz group treated with PBS. It has been reported that TUDCA's actions in microglia cells can be mediated by direct activation of its Takeda G protein-coupled receptor 5 (TGR5), which results in increased intracellular cAMP levels, inducing the expression of anti-inflammatory markers and reducing proinflammatory ones^47^. In addition, the previously reported ability of TUDCA involved in reducing Aβ production may also have contributed to the reduction of microglia activation^21,23,24^.

Interestingly, Clarke and colleagues reported that AgRP and NPY expression remained unchanged in mice that received ICV injection of βA oligomers, and that were pretreated with minocycline, an antibiotic that prevents microglial activation, suggesting that Aβ oligomers probably affect microglial cells, inducing the secretion of inflammatory factors involved with increased expression of AgRP and NPY^10^. Thus, we believe that the effects of TUDCA in mitigating hypothalamic inflammation are involved, at least in part, with the normalization of the expression of orexigenic neuropeptides in Stz + TUDCA mice.

Still, it is well established that hypothalamic inflammation is involved with leptin resistance^48,49^. Under normal conditions, leptin causes an anorexigenic response via activation of leptin receptor signaling pathways in hypothalamic neurons. Leptin binding to its receptor (Ob-Rb) induces activation of JAK2, receptor dimerization, and JAK2-mediated phosphorylation of intracellular part of Ob-Rb, followed by phosphorylation and activation of STAT3. Activated STAT3 dimerizes, translocates to the nucleus and modulates target genes expression, including NPY and POMC^44^. This effect reduces appetite and increases EE, which contribute to the maintenance of energy homeostasis^44^.

We observed in Stz mice reduced levels of JAK2 and STAT3 phosphorylation, as well as higher acute food intake after stimulation with leptin, suggesting impaired hypothalamic leptin signaling. Interestingly, compared to Stz mice, Stz + TUDCA group displayed higher levels of p-JAK2 and p-STAT3, and lower food intake after leptin load. Corroborating our data, a previous study reported that TUDCA increases leptin sensitivity in the leptin-deficient (ob/ob) and high fat diet-induced obese mice, acting as a leptin sensitizer agent^50^. These findings suggest that the action of TUDCA in decreasing inflammation and promoting the preservation of leptin signaling in the hypothalamus may contributes to the normalization of the eating behavior observed in these animals. Furthermore, the higher EE observed in animals treated with TUDCA in the dark cycle (feeding period), might be the result of the preservation of hypothalamic leptin signaling in these animals, since through the JAK2/STAT3 pathway leptin activates EE^51^.

BAT also plays an important role in increasing EE, through its thermogenic capacity^52^. This tissue contains a large number of mitochondria, which act as heat generators through the UCP1^52,53^. However, even observing an increase in BAT weight of Stz + TUDCA group, compared to Stz mice, RT-qPCR analysis showed that the expression levels of BAT-specific genes related to heat generation, including UCP1, DIO2, PPARGC1α, CIDEA, PRDM16, COX7A1 and COX8B remained statistically equal between groups. These data suggest that BAT thermogenesis is not directly involved in ameliorated EE observed in Stz animals treated with TUDCA.

Another important point is the ability of TUDCA to enhance beige adipocytes differentiation in vitro and inguinal white adipose tissue (iWAT) browning in vivo, through its chemical chaperone action, attenuating ER stress^54^. Considering that beige adipocytes are specialized to dissipate excess chemical energy through UCP1-mediated heat formation^55^, their activation has been recognized as a therapeutic target to increase EE^54^. Besides that, it has previously been reported that ICV injection of βA oligomers induces adipose tissue inflammation^10^. Since inflammation and ER stress are closely related^56,57^, TUDCA, by reducing ER stress in this tissue, could be contributing to the energy balance, via activation of iWAT browning. Supporting this idea, bile acid receptor TGR5 activation in WAT participates in the browning process by increasing β-oxidation and improving mitochondrial function^58^. Thus, we speculate that TUDCA treatment could also be involved with the enhanced beige adipocytes differentiation, impacting EE of Stz + TUDCA group. However, further studies are still needed to understand weather TUDCA modulates browning in an AD mice model.

Taken together, we believe that the increase in NPY and AgRP gene expression observed in Stz group may be involved with failures in hypothalamic leptin signaling, probably due to hypothalamic inflammation, so that leptin does not satisfactorily promote inhibition of this neuronal population. In this regard, treatment with bile acid TUDCA exert beneficial effects on the energetic homeostasis of this model, attenuating the expression of inflammatory markers and preserving the leptin sensitivity in the hypothalamus, resulting in the improvement of body weight, adiposity and EE.

Results of clinical studies carried out in patients with amyotrophic lateral sclerosis have pointed out that TUDCA have high safety profile in humans, in addition to good absorption after oral administration and penetration into the cerebrospinal fluid^20^. Therefore, considering the beneficial effects of TUDCA on the energy metabolism of experimental AD mice model, our results expand the understanding of the actions and pathways triggered by this bile acid, contributing to the validation of TUDCA’s clinical application in AD.

## Supplementary Information

Supplementary Information.
