# Supplementary material for: Energy homeostasis deregulation is attenuated by TUDCA treatment in streptozotocin-induced Alzheimer’s disease mice model
Source: Sci Rep. 2021 Sep 13;11:18114. doi: 10.1038/s41598-021-97624-6 (PMC8437965; doi:10.1038/s41598-021-97624-6)
Supplement: Supplementary file 1 — Supplementary Information. [file 41598_2021_97624_MOESM1_ESM.docx]

**Supplementary Information**

**Energy homeostasis deregulation is attenuated by TUDCA treatment in streptozotocin-induced Alzheimer’s disease mice model**

Lucas Zangerolamo^1^, Carina Solon^2^, Gabriela M. Soares^1^, Daiane F. Engel^2^, Licio A. Velloso^2^, Antonio C. Boschero^1^, Everardo M. Carneiro^1^, Helena C. L. Barbosa^1*^

^1^ Obesity and Comorbidities Research Center, Department of Structural and Functional Biology, University of Campinas, UNICAMP, Campinas, Sao Paulo, Brazil.

^2^ Laboratory of Cell Signaling, Obesity and Comorbidities Research Center, University of Campinas, UNICAMP, Campinas, Sao Paulo, Brazil.

***Correspondence:** Helena Cristina L. Barbosa, Obesity and Comorbidities Research Center, University of Campinas, UNICAMP, Campinas, Sao Paulo, CEP: 13083-864, Brazil.

Tel.: +55 19 3521 0011.

E-mail address: [bsampaio@unicamp.br](mailto:bsampaio@unicamp.br)

**Full unedited gel from Figure 4 (D-F)**

**Groups: Ctl (1), Stz (2), Stz+TUDCA (3)**

**Membrane 01**


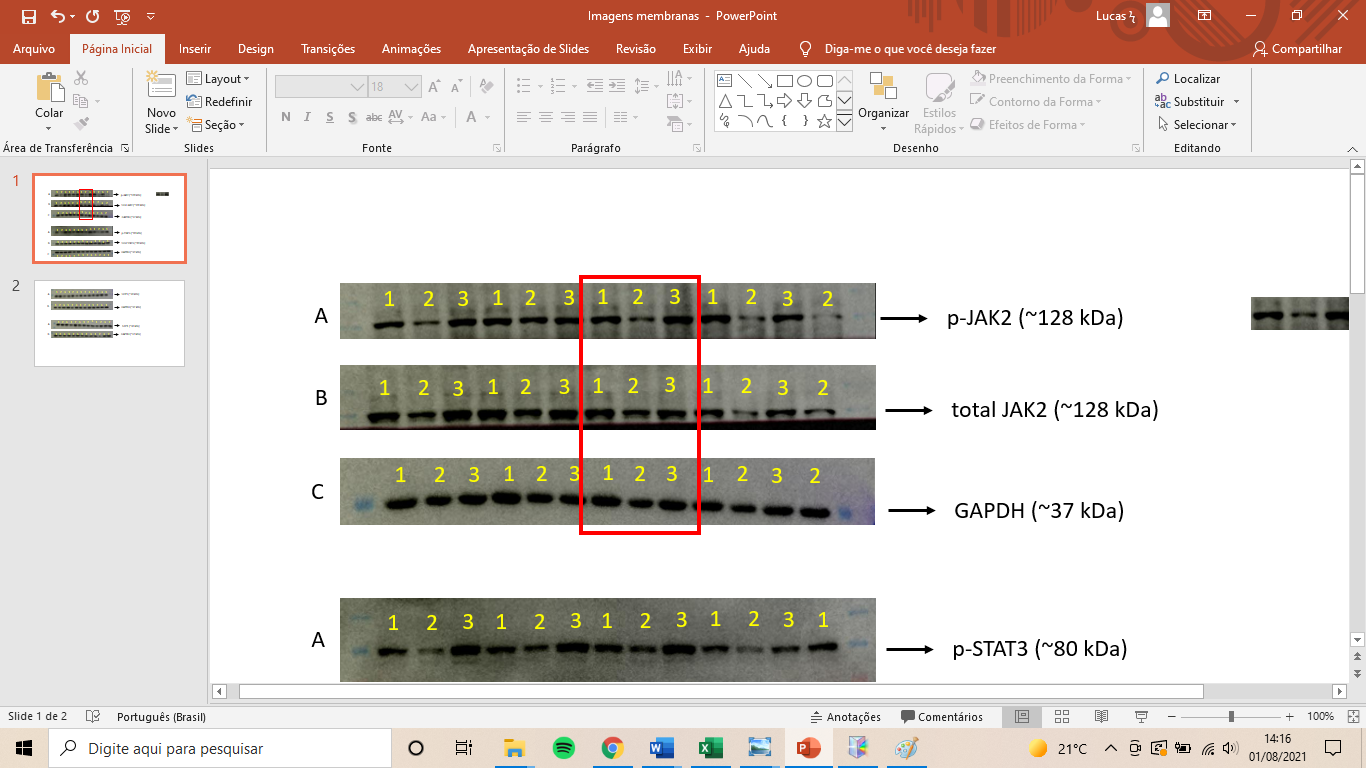


Supplementary Figure 1. Full scan of the entire original gel incubated with p-JAK2 (Tyr1007/Tyr1008) (A), total JAK2 (B), and GAPDH (C) antibody. Red Box indicate the representative image shown in Fig 4D. The samples were transferred to nitrocellulose membranes in this sequence: Ctl (1), Stz (2) and Stz+TUDCA (3).

**Membrane 02**


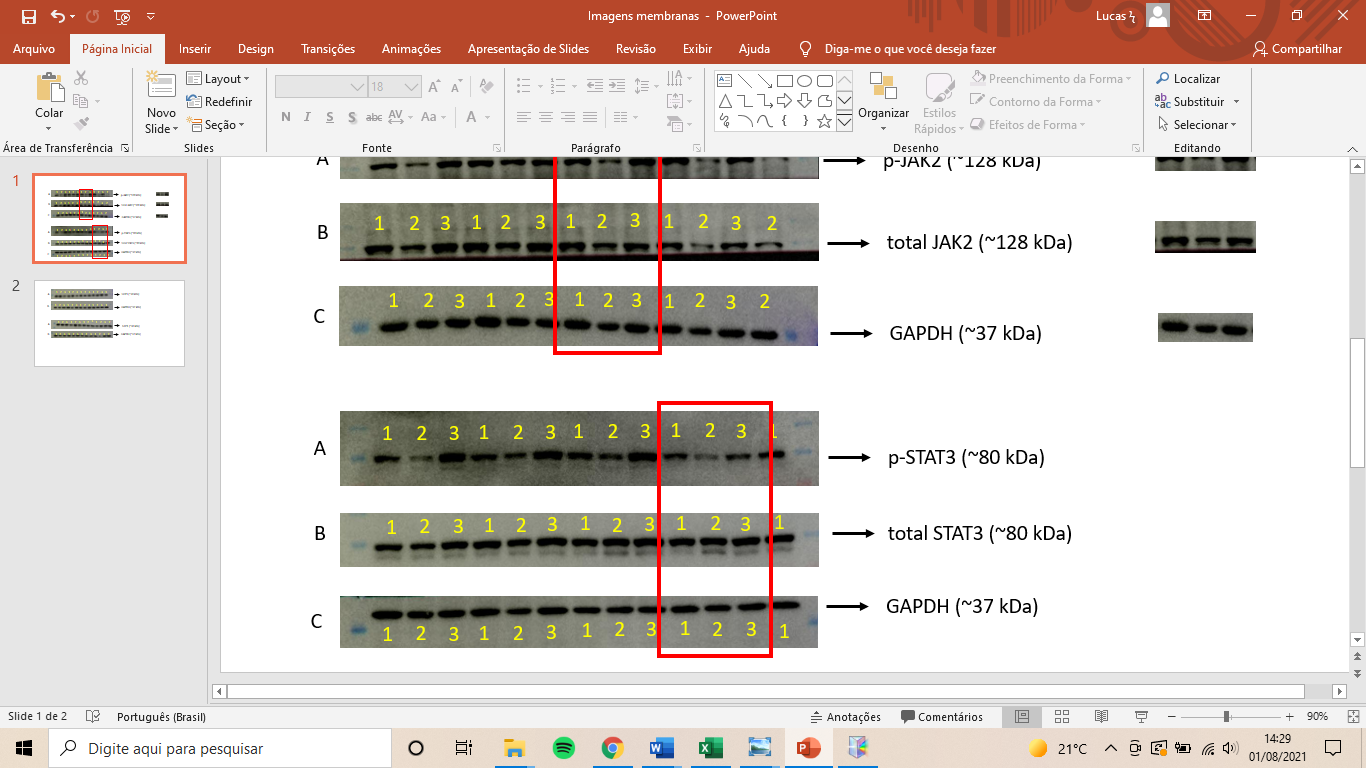


Supplementary Figure 2. Full scan of the entire original gel incubated with p-STAT3 (Tyr705) (A), total STAT3 (B), and GAPDH (C) antibody. Red Box indicate the representative image shown in Fig 4D. The samples were transferred to nitrocellulose membranes in this sequence: Ctl (1), Stz (2) and Stz+TUDCA (3).

**Full unedited gel from Figure 5 (H)**

**Groups: Ctl (1), Stz (2), Stz+TUDCA (3)**

**Membrane 03**


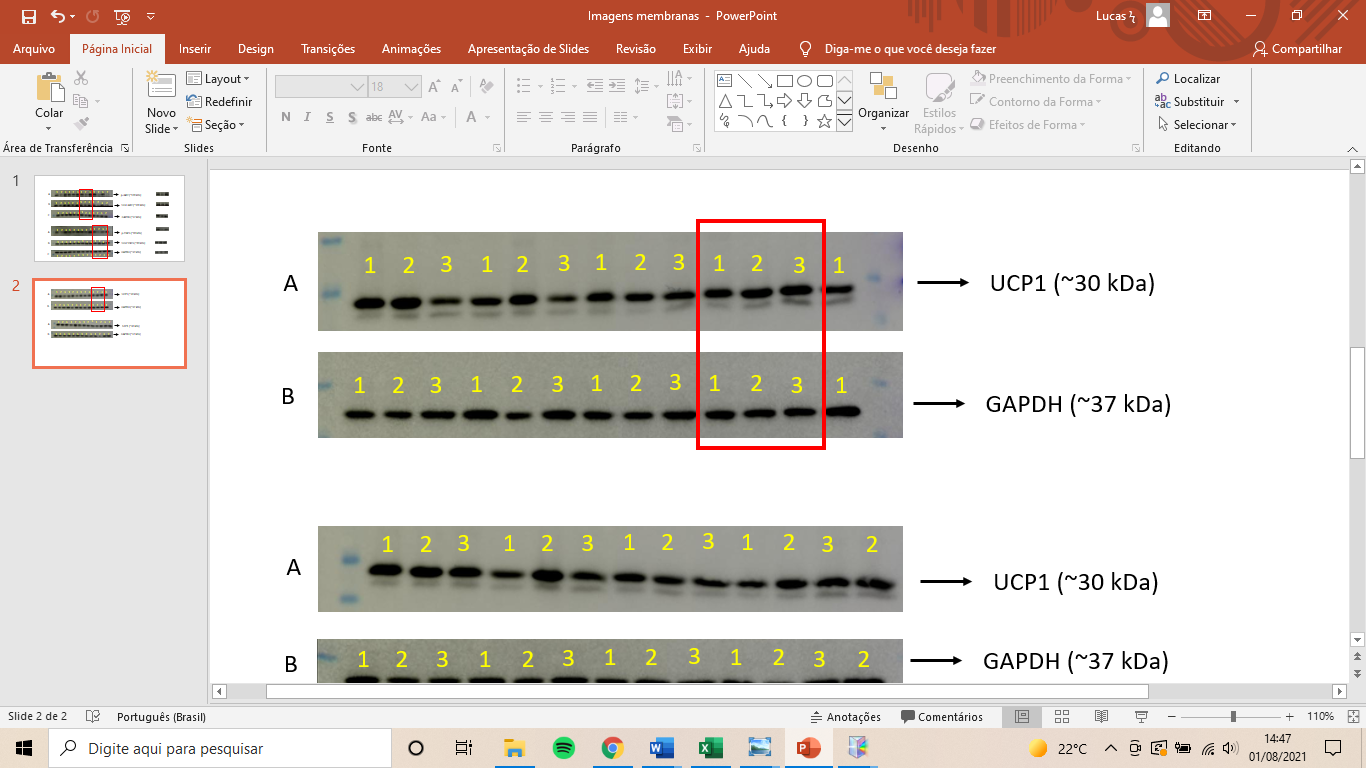


Supplementary Figure 3. Full scan of the entire original gel incubated with UCP1 (A) and GAPDH (B) antibody. Red Box indicate the representative image shown in Fig 5H. The samples were transferred to nitrocellulose membranes in this sequence: Ctl (1), Stz (2) and Stz+TUDCA (3).

**Membrane 04**


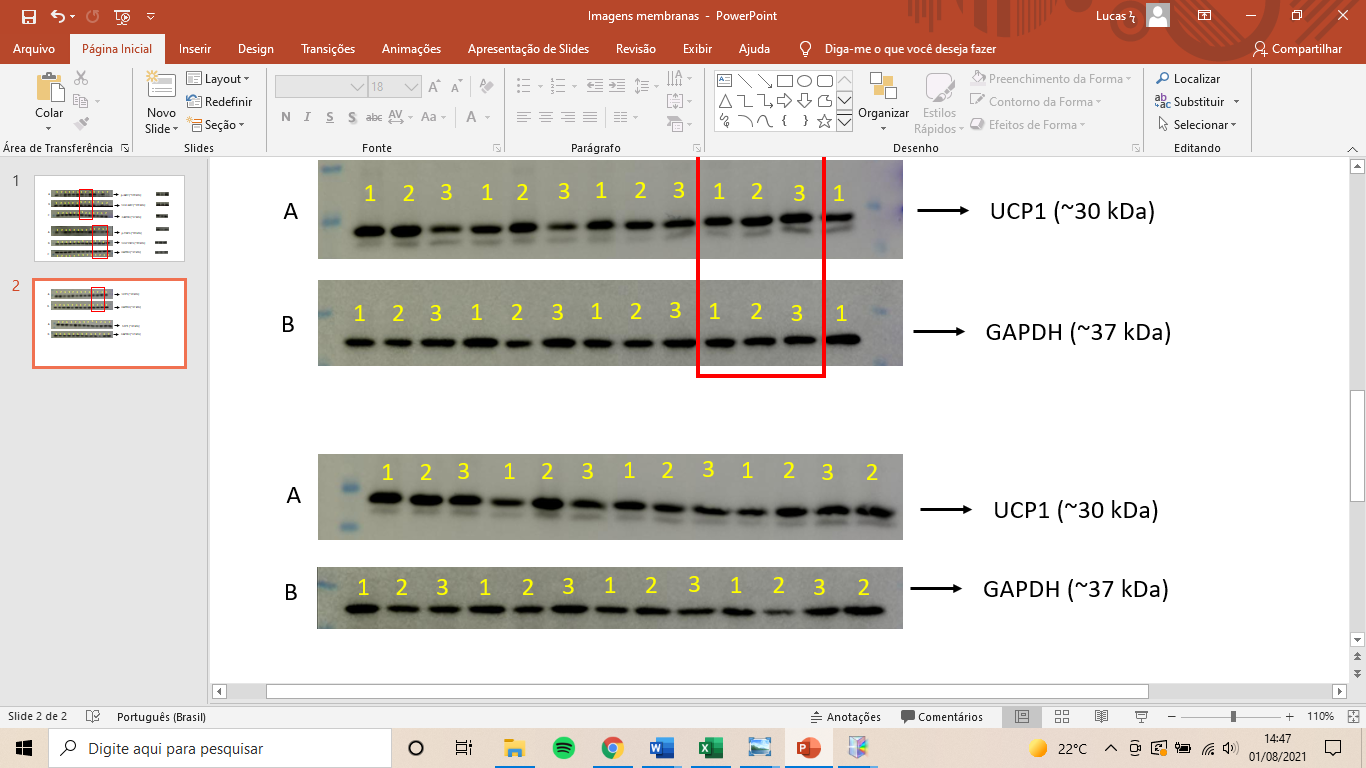


Supplementary Figure 4. Full scan of the entire original gel incubated with UCP1 (A) and GAPDH (B) antibody. The samples were transferred to nitrocellulose membranes in this sequence: Ctl (1), Stz (2) and Stz+TUDCA (3).
